# 1,25-Dihydroxyvitamin D inhibits hepatic diacyglycerol accumulation and ameliorates metabolic dysfunction in polycystic ovary syndrome rat models

**DOI:** 10.3389/fphar.2023.1077014

**Published:** 2023-04-05

**Authors:** Xin Yuan, Jianshu Yang, Danlin Sun, Kaiming Luo, Xiaohong Jiang, Long Wang, Shoukui Xiang, Yijie Jiang, Kele Ge, Zhiyang Zhou, Bowen Li, Fei Hua

**Affiliations:** ^1^ Department of Endocrinology, The First People’s Hospital of Changzhou, Changzhou, China; ^2^ Health Management Center, The First Affiliated Hospital of Soochow University, Suzhou, China; ^3^ Department of Neurosurgery, The First People’s Hospital of Changzhou, Changzhou, China; ^4^ Clinical Medical Research Center, The First People’s Hospital of Changzhou, Changzhou, China; ^5^ Department of Oncology, The First People’s Hospital of Changzhou, Changzhou, China; ^6^ LipidALL Technologies Company Limited, Changzhou, China

**Keywords:** vitamin D, polycystic ovary syndrome, metabolic dysfunction, lipidomics, diacyglycerol

## Abstract

**Introduction:** We aimed to evaluate the influence of 1,25-dihydroxyvitamin D (1,25(OH)_2_D) on metabolic dysfunction and elucidate its underlying mechanism using a rat model of polycystic ovary syndrome (PCOS).

**Methods:** Twenty-four Sprague-Dawley rats were randomly divided into four groups: control group (CON, 2 ml/kg of oral 0.5% CMC), 1,25VD group (oral 0.5% CMC and 2.5 ug/kg intraperitoneal 1,25(OH)_2_D), PCOS group (1 mg/kg oral letrozole), PCOS+1,25VD group (1 mg/kg oral letrozole orally 2.5 ug/kg intraperitoneal 1,25(OH)_2_D). The treatments were administered for 8 weeks. Body weight, estrus cycle, insulin tolerance, and oral glucose tolerance of the rats in the different groups were assessed. The rats were euthanized at the 8th weeks, and plasma, ovarian, and liver samples were collected and analyzed. The hepatic lipid profile was characterized using HPLC/MRM.

**Results:** Letrozole-induced PCOS rats exhibited increased weight, insulin resistance, postprandial glucose abnormalities, and dyslipidemia. Compared with the PCOS group rats, the PCOS+1,25VD group rats showed reduced body weight, increased sensitivity to insulin, decreased postprandial glucose, and elevated levels of high-density lipoprotein cholesterol. Moreover, abnormally increased liver concentrations of total diacylglycerol (DG) and DG species in the PCOS rats were reversed by treatment with 1,25(OH)_2_D. Additionally, hepatic DG and insulin sensitivity were correlated.

**Conclusion:** 1,25(OH)_2_D inhibited hepatic DG accumulation and ameliorated metabolic dysfunction in PCOS rat models.

## 1 Introduction

Polycystic ovary syndrome (PCOS) is a commonly diagnosed endocrine disease, with a prevalence of PCOS approximately 4%–20% worldwide (Bozdag et al., 2016; Skiba et al., 2018). Hyperandrogenism, oligomenorrhea, and polycystic ovarian morphology are the most common clinical characteristics of PCOS (Bozdag et al., 2016; Skiba et al., 2018). PCOS may also be accompanied by other metabolic dysfunctions, including obesity, insulin resistance, and dyslipidemia (EA-SPcwg, 2004). The risk of developing cardiovascular disorders, type 2 diabetes, non-alcoholic fatty liver disease and endometrial carcinoma is more in women with PCOS than in healthy subjects (Barry et al., 2014; Rocha et al., 2017; Osibogun et al., 2020). The pathogenesis of PCOS is yet to be fully elucidated, and therefore, there is no effective cure. Current therapies have been designed to ameliorate heterogeneous clinical symptoms (Goodarzi et al., 2011; Walters et al., 2018). Hence, the development of novel and effective anti-PCOS therapies is in high demand.

Vitamin D deficiency is a global health concern (van Schoor and Lips, 2017). Vitamin D functions as a key regulator in maintaining calcium homeostasis and bone development, and accumulating evidence suggests that it also has wide extra-skeletal functions. Decreased vitamin D levels are reportedly associated with multiple disorders such as autoimmune diseases, cancers, cardiovascular diseases, and type 2 diabetes (Bouillon et al., 2008). Vitamin D deficiency is also common in PCOS and may be associated with the development of several of its clinical features (Trummer et al., 2018; Eftekhar et al., 2020). However, the role of vitamin D deficiency in the progression of PCOS needs to be further explored. Existing studies have produced contradictory results on the application of vitamin D as a supplement in treating PCOS (Plum and DeLuca, 2010; Pergialiotis et al., 2017). Therefore, it remains unclear whether vitamin D supplementation could play a positive role in improving the therapeutic efficacy of current anti-PCOS therapies.

Exposure to letrozole, an aromatase inhibitor, reportedly prevents the conversion of testosterone to estradiol, which further elevates the serum androgen levels and leads to polycystic ovarian morphology and other metabolic disorders in a rat model (Stener-Victorin et al., 2020). 1,25-dihydroxyvitamin D [1,25(OH) _2_D], an active form of vitamin D, exerts its biological function by interacting with vitamin D receptors (Plum and DeLuca, 2010). Herein, we aimed to assess whether 1,25(OH)_2_D exerted beneficial effects on metabolic dysfunction in a letrozole-induced PCOS rat model. We also evaluated the hepatic lipid content of diacylglycerol (DG) and triacylglycerol (TG) using a lipidomics strategy. The results of the current study may provide novel evidence for the application of vitamin D supplements as a cost-effective therapeutic strategy in PCOS management.

## 2 Material and methods

### 2.1 Animals and treatments

Twenty-four (4-5-week-old) rats, weighing 78–97 g, were purchased from the Shanghai Family Planning Research Institute, Shanghai, China. The rats (six per cage) were housed under the following conditions: 12 h light/dark cycle, 21°C–22°C, 50%–60% humidity, and free access to food and distilled water. The Soochow University Animal Care and Use Committee (Suzhou, China) approved this study (No: Date of approval). The rats were divided into the following groups: 1) control group (CON, N = 6) where rats were intragastrically administered 0.5% carboxymethyl cellulose (CMC) at a daily dose of 2 ml/kg; 2) 1,25(OH)_2_D supplemental group (1,25VD, N = 6) where rats received 0.5% CMC gavage daily and an intraperitoneal (i.p.) injection of 2.5ug/kg of 1,25(OH)_2_D (Sigma Aldrich, St. Louis, MO, United States) dissolved in a 0.5% ethanol and 95.5% culture medium solution every 2 days (Yin et al., 2012); 3) Letrozole group (PCOS, N = 6) where rats were intragastrically administered 1 mg/kg of letrozole (Hengrui Medicine Co., Jiangsu, China) dissolved in 0.5% CMC solution daily and i. p. injection of the vehicle every 2 days; and 4) Letrozole + 1,25(OH)_2_D supplemental group (PCOS+1,25VD, N = 6) where rats received 1 mg/kg of letrozole gavage and 2.5 ug/kg of i.p. 1,25(OH) _2_D injection every 2 days.

**FIGURE 1:**
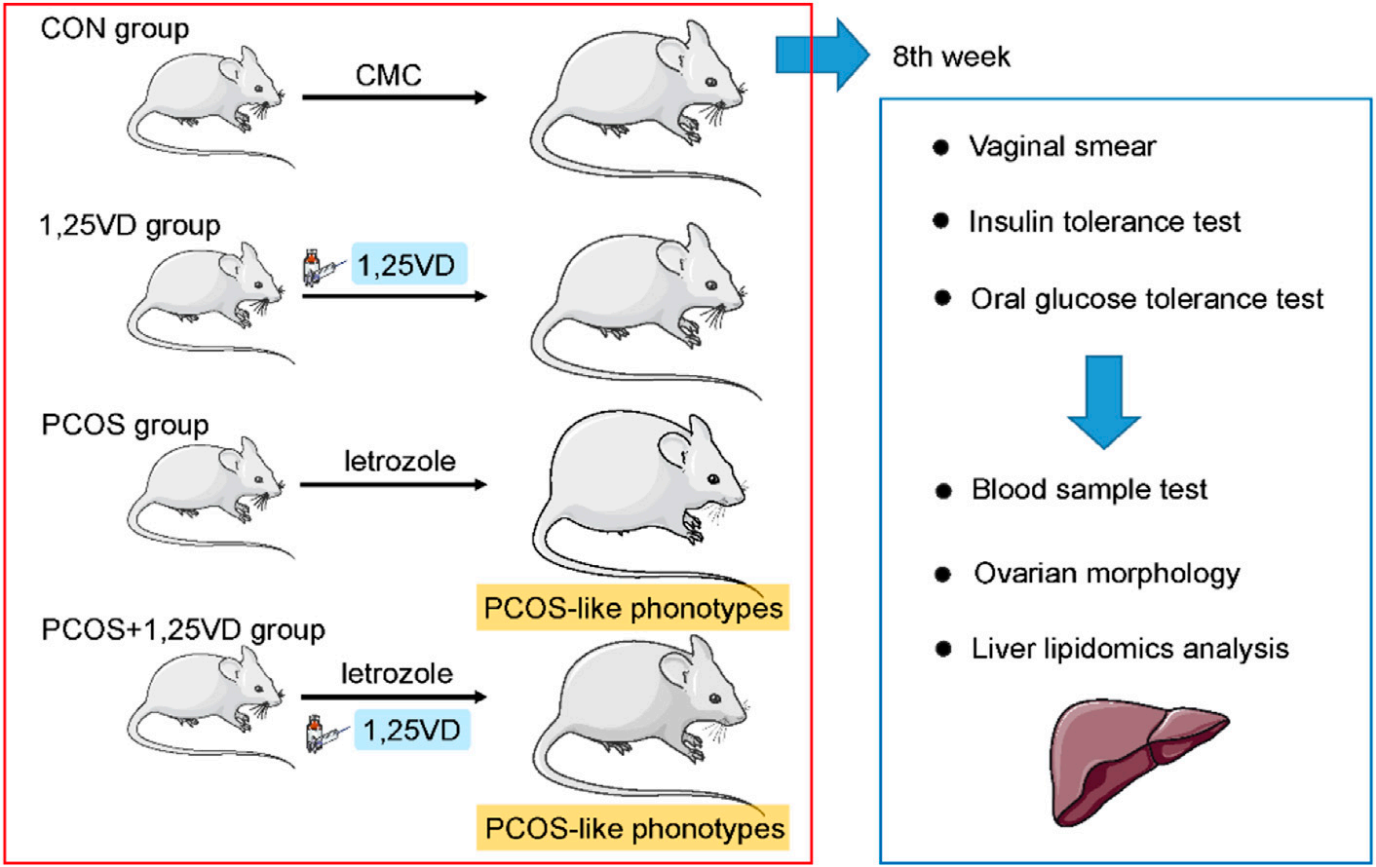
Schematic diagram of the animal study design. Rats were randomly assigned to the 1,25-dihydroxyvitamin D_3_ supplementation or vehicle group. Vaginal smear, insulin tolerance test and oral glucose tolerance test were conducted during the 8th weeks. Serum samples were collected for the evaluation of the hormone and lipid levels. Ovarian morphology was examined to verify the establishment of PCOS in the models. Hepatic samples were subjected to lipidomic profiling.

The treatments were administered for 8 weeks. Rats were weighed twice per week. Vaginal smears were obtained daily during the 8th weeks to validate the induction of PCOS models. Insulin tolerance and oral glucose tolerance tests were completed on separate days of the 8th weeks. After the 8th weeks, the animals kept fasting overnight. Subsequently, they were anesthetized with chloral hydrate (3 ml/kg intraperitoneal). The blood samples were obtained from the abdominal aorta and centrifuged at 4500 rpm for 10 min at 4°C to collect the serum. Bilateral ovary and liver samples were dissected and weighed. One ovary was treated with formalin (10% solution) to determine morphological changes; the other ovary was stored at −80°C for the lipidomics analysis (Sun et al., 2017) (Figure 1). The present study has been approved by Soochow University Animal Care and Use Committee (NO.20210907).

### 2.2 Insulin tolerance test

The rats were kept fasting overnight. Thereafter, the rats were intraperitoneally administered insulin (1 IU/kg). Blood samples were collected from the tail vein. Blood glucose levels at 0, 15, 30, 60, and 90 min after insulin administration were measured using a blood glucometer (ASCENSIA Diabetes Care).

### 2.3 Oral glucose tolerance test

The rats were intragastrically administered glucose (2 g/kg) after overnight fasting. Blood samples were collected from the tail vein. Blood glucose levels at 0, 15, 30, 60, 90, and 120 min after glucose administration were measured using a blood glucometer (ASCENSIA diabetes care).

### 2.4 Blood samples tests

Serum samples were collected and enzyme-linked immunosorbent assay (ELISA) kits (Sangon Biotech) were used to analyze testosterone, luteinizing hormone, and follicle-stimulating hormone levels. Serum 25-hydroxyvitamin D [25(OH)D] levels were measured using radioimmunoassay (Roche Diagnostics). Serum triglyceride, total cholesterol, high-density lipoprotein cholesterol (HDL-c), and low-density lipoprotein cholesterol (LDL-c) levels were analyzed using an automatic biochemical analyzer (Beckman Coulter AU5800).

### 2.5 Histology study

Histological studies were performed as previously described (Choubey et al., 2019). Ovarian samples were fixed using formalin, embedded in paraffin, and stained with hematoxylin-eosin (H&E) solution. Vaginal smears were stained with Giemsa solution (Upadhyay et al., 2021). A sterile, saline-soaked cotton swab was inserted daily into the rat’s vagina at 8:00 a.m., gently rotated clockwise, and then smeared onto a clean slide. The smear was allowed to dry and stained with Giemsa solution for 15 min. The type and proportion of cells observed under the light lens were used to determine the stage of the estrous cycle in the rats as follows: 1) Diestrus: Leukocyte predominance with occasional nucleated epithelial cells; 2) Pre-estrus: predominance of oval-shaped nucleated dermal cells with occasional leukocytes and non-nucleated keratinized epithelial cells; 3) Estrous: Predominance of lamellar, non-nucleated, keratinized epithelial cells and a few nucleated epithelial cells and leukocytes; and 4) Metestrus: Nucleated keratinized epithelial cells and leukocytes with a few nucleated epithelial cells.

### 2.6 Isolation of the liver lipids

The lipids were isolated from 30 mg of rat liver using the modified Bligh and Dyer’s method (Lam et al., 2016). Liver samples were homogenized using 750 µl of chloroform, methanol, and MilliQ H2O solution (3:6:1). The homogenates were centrifuged for 60 min (1500 rpm at 4°C). Thereafter, thee samples were treated with deionized H_2_O (350 µl) and chloroform (250 µl). Subsequently, the samples were centrifuged for 5 min (12000 rpm at 4°C). After centrifugation, the lower phase was transferred to a new tube. Chloroform (500 µl) was added to the aqueous phase to extract the remaining lipids. Finally, the lipids were collected, dried using SpeedVac, and stored at −80°C until needed.

### 2.7 Lipidomics

All lipidomic analyses were performed at LipidALL Technologies (Changzhou, China) using an Agilent 1290 II UPLC coupled with a Sciex QTRAP 6500 PLUS (Lam et al., 2021). Individual lipid species were quantified by referencing spiked internal standards. d9-PC32:0 (16:0/16:0), d9-PC36:1p (18:0p/18:1), d7-LPC18:1, d7-PE33:1 (15:0/18:1), d9-PE36:1p (18:0p/18:1), d7-LPE18:1, d31-PS, C17-LPS, d7-PA33:1 (15:0/18:1), C17 LPA, d7-PG33:1 (15:0/18:1), C17:1-LPG, d7-PI33:1 (15:0/18:1), C17-LPI, d5-CL72:8 (18:2)4, C17-SL and C14-BMP were purchased from Avanti Polar Lipids (AL, United States). GM3-d18:1/18:0-d3 cells were obtained from Matreya LLC (PA, United States). A modified version of the reverse-phase HPLC/MRM was used for the quantification of glycerol lipids (DG and TG). A 2.6 µm Phenomenex Kinetex-C18 column (i.d. 4.6 × 100 mm) was applied for 17 min (flow rate: 170 µl) to separate the neutral lipids using an isocratic mobile phase (including chloroform:methanol:0.1 M ammonium acetate in the ratio of 100:100:4 [v/v/v]). Short, medium, and long-chain TGs were quantified using TG (14:0)3-d5, TG (16:0)3-d5, and TG (18:0)3-d5 (CDN isotopes). DGs were determined using the internal standards d5-DG17:0/17:0 and d5-DG18:1/18:1 (Avanti Polar Lipids). Free fatty acids were determined using the internal standards d31-16:0 (Sigma-Aldrich) and d8-20:4 (Cayman Chemicals).

### 2.8 Statistical analysis

All data are expressed as mean ± standard deviation. Statistical analyses were performed using SPSS (version 26.0; SPSS Inc.). Comparisons between two groups were performed using Student’s t-test. Comparisons among multiple groups were performed using analysis of variance (ANOVA) with Tukey’s *post hoc* tests. Correlation analysis was performed using Pearson’s correlation coefficient. Statistical significance was set as *p* < 0.05.

## 3 Results

### 3.1 Establishment of letrozole-induced PCOS rat models

**FIGURE 2:**
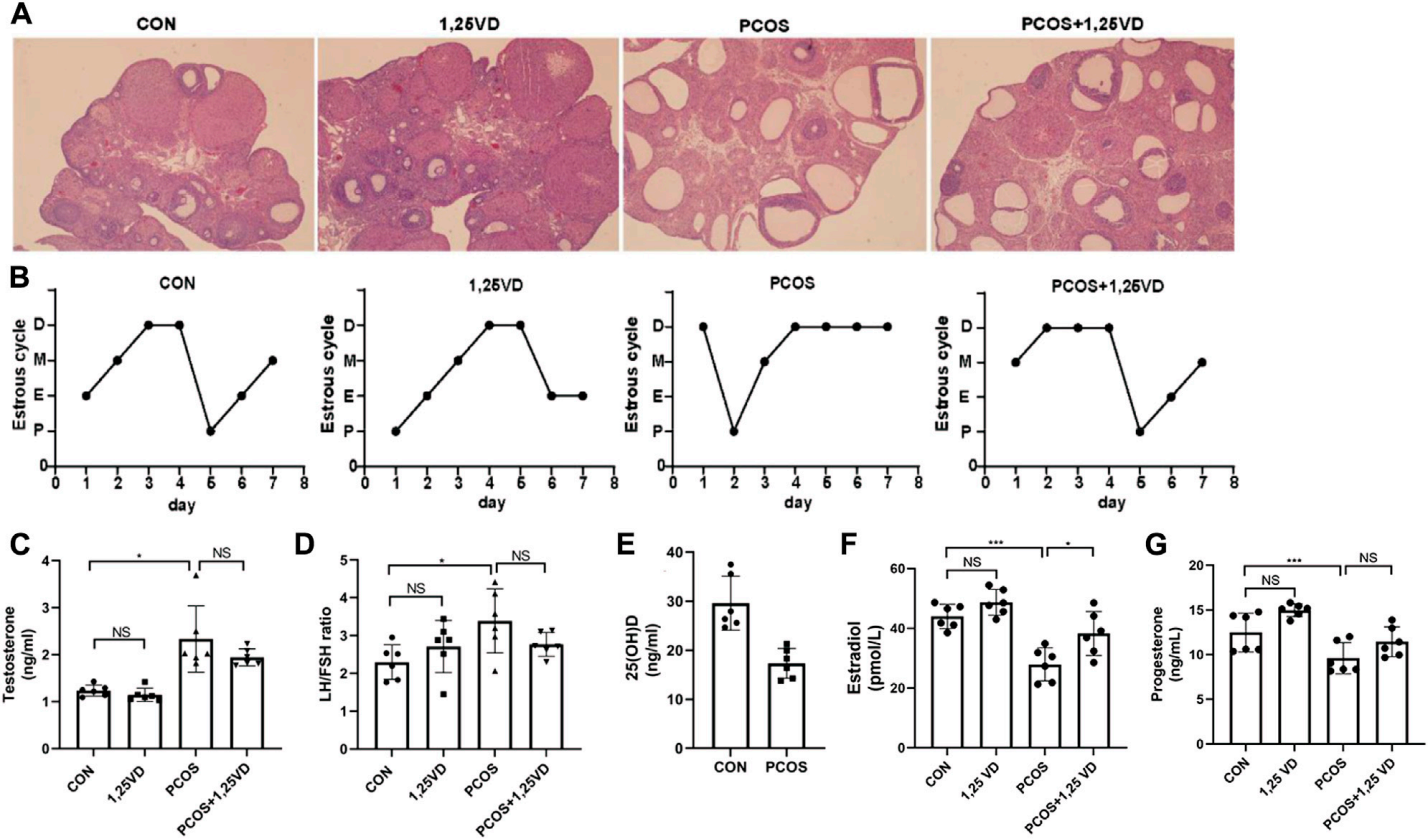
Ovarian morphology **(A)**, estrus cycle **(B)**, serum testosterone levels **(C)**, LH/FSH ratio **(D)**, 25(OH)D **(E)**, estradiol **(F)** and progesterone **(G)** of the different groups. D: Diestrus, M: Metaoestrus, E: Estrus, P: Proestrus, LH: Luteinizing hormone, FSH: Follicle stimulating hormone. *p < 0.05, **p < 0.01, ***p < 0.001.

First, a histological analysis was performed to characterize the letrozole-induced PCOS rat model. Polycystic ovarian morphology was observed in letrozole-treated rats, with few normal follicles at different developmental stages and corpus luteum due to abnormal follicle development and anovulation (Figure 2A). Furthermore, as shown in Table 1, the numbers of primordial/primary follicles, secondary follicles, tertiary follicle, cysts were all decreased and the number of cystic follicles was markedly increased in PCOS group, suggesting that the PCOS model has been successfully established. The vaginal smear analysis proved that letrozole-treated rats tended to stay in the diestrus phase and had no estrus cycle (Figure 2B). Moreover, compared with the control group, letrozole treatment led to a nearly two-fold increase in the serum testosterone levels and an elevated LH/FSH ratio. Meanwhile, 1,25(OH)_2_D treatment had no significant effect on testosterone and the LH/FSH ratio in the PCOS rats (Figures 2C, D). Furthermore, the PCOS group showed reduced serum 25(OH)D levels compared with the CON group (Figure 2E). Furthermore, the levels of estradiol and progesterone were all decreased in PCOS group and increased by 1,25(OH)2D treatment (Figures 2F, G).

**TABLE 1:** Description of the ovarian morphology.

	Control	Control+1,25 VD	PCOS	PCOS+1,25 VD
Primordial/primary follicles	2.17 ± 0.75	2.00 ± 0.63	0.83 ± 0.75***	1.50 ± 0.84##
Secondary follicles	1.83 ± 0.75	1.33 ± 0.82	0.50 ± 0.84**	1.00 ± 1.26##
Tertiary follicles	0.67 ± 0.52	0.83 ± 0.75	0.33 ± 0.52**	0.50 ± 0.84
Corpus luteum	3.17 ± 1.72	3.5 ± 0.84	0.17 ± 0.41***	0.50 ± 0.84#
cystic follicles	0.33 ± 0.52	0.50 ± 0.55	9.67 ± 2.34***	8.83 ± 4.17

**p < 0.01 v.s. control, ***p < 0.001 v. s. control #*p* < 0.05 v.s PCOS, #*p* < 0.01 v.s PCOS.

### 3.2 Supplementation of exogenous 1,25(OH)_2_D_3_ prevents weight gain, insulin resistance and HDL-c decrease in PCOS rats

**FIGURE 3:**
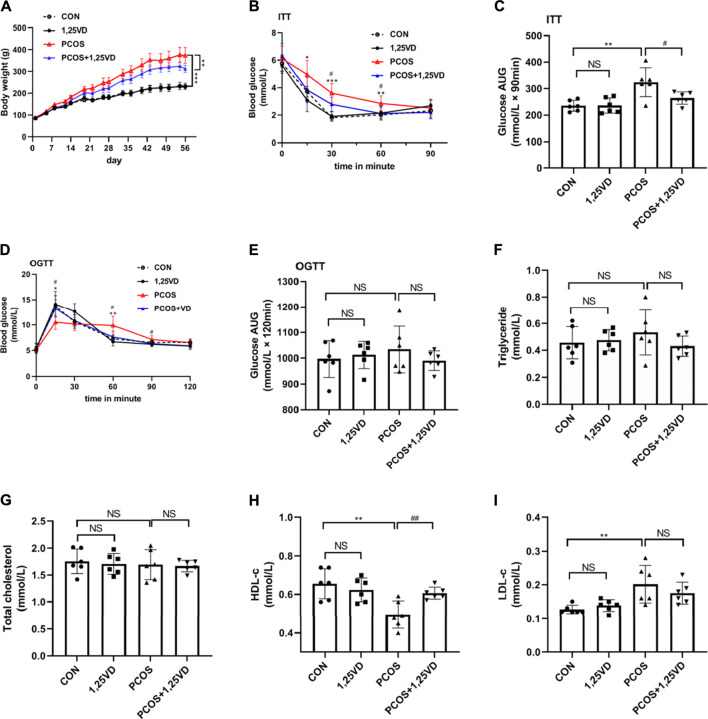
Comparison of the body weight **(A)**, ITT **(B)**, area under the curve (AUC) for glucose in ITT **(C)**, OGTT **(D)**, AUC for glucose in OGTT **(E)**, serum triglyceride levels **(F)**, total cholesterol levels **(G)**, HDL-c levels **(H)** and LDL-c levels **(I)** among the study groups. *: PCOS *vs*. CON *p* < 0.05, **: PCOS *vs*. CON *p* < 0.01, ***: PCOS *vs*. CON *p* < 0.001, #: PCOS+1,25VD *vs*. PCOS *p* < 0.05, ##: PCOS+1,25VD *vs*. PCOS *p* < 0.01. HDL-c: High density lipoprotein cholesterol, LDL-c: Low density lipoprotein cholesterol, ITT, OGTT.

At the beginning of the study, there were no significant differences in the body weight among the four groups. After 8 weeks of treatment, the weight of the rats in the PCOS group was significantly higher than that of the control rats (Figure 3A); However, treatment with 1,25(OH)_2_D significantly decreased the body weight of PCOS rats (Figure 3A). During the insulin tolerance test (ITT), there was a significant increase in glucose levels in PCOS rats compared with the control rats after 15, 30, and 60 min, whereas, the PCOS+1,25VD group showed reduced glucose levels after 30 and 60 min. The area under the curve (AUC) exhibited the same trend. Insulin resistance was observed in the PCOS group, whereas 1,25(OH)_2_D increased insulin sensitivity (Figures 3B, C). During the oral glucose tolerance test (OGTT), glucose levels decreased in PCOS rats at 15 min and were elevated at 60 min compared to the control rats. The glucose level in the PCOS + 1,25VD group was higher at 15 min than that in the PCOS group; Thereafter, the glucose levels decreased significantly after 60 min and 90 min (Figure 3D). The AUC, circulating TGs, and total cholesterol showed no significant differences among the four groups (Figures 3E–G). The circulating HDL-c levels decreased, while the circulating LDL-c levels increased in the PCOS group. Conversely, the circulating HDL-c levels increased in the PCOS+1,25VD group (Figures 3H, I).

### 3.3 1,25(OH)_2_D_3_ reduces hepatic DG content in PCOS rats

**FIGURE 4:**
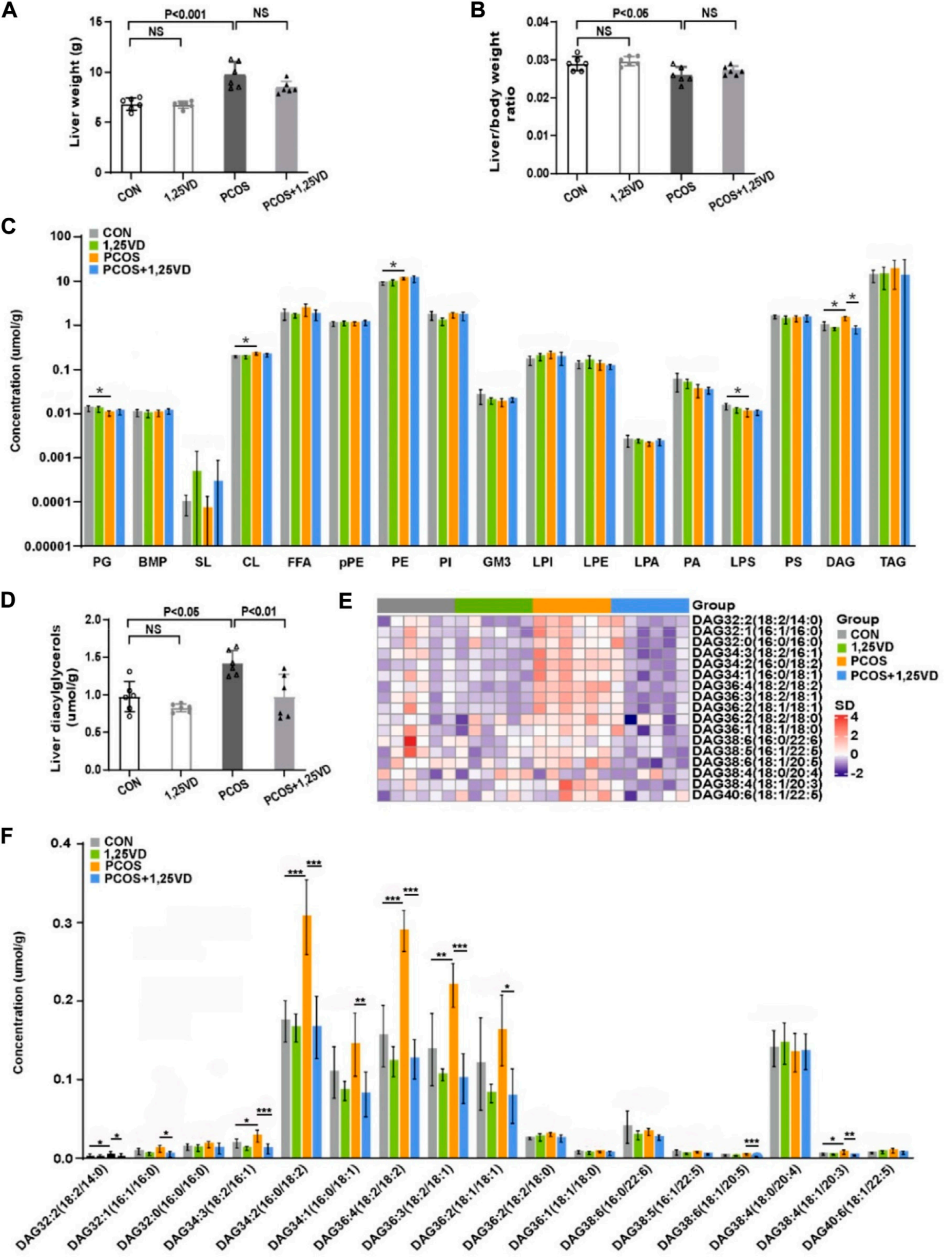
Comparison of the liver weight **(A)**, liver/body weight ratio **(B)** and hepatic lipid profile **(C–F)** among the study groups. PG: Phosphatidylglycerols, BMP: Bis (monoacylglycerol) phosphate, SL: Sulfatides, CL: Cardiolipins, FFA: Free fatty acids, pPE: PlasmalogenPE, PE: Phosphatidylethanolamines, PI: Phosphatidylinositols, GM3: Monosialogangliosides, LPI: Lyso-PI, LPE: Lyso-PE, LPA: Lyso-PA, PA: Phosphatidic acids, LPS: Lyso-PS, PS: Phosphatidylserines, DAG: Diacylglycerols, TAG: Triaclglycerols. *p < 0.05, **p < 0.01, ***p < 0.001.

After 8 weeks of treatment, the liver weight of the rats in the PCOS group was significantly higher than that of the control rats (Figure 4A), whereas the liver/body weight ratio in the PCOS group was lower than that in the control group (Figure 4B). Liver weight and liver/body weight ratio showed no significant differences between the PCOS and PCOS+1,25VD groups (Figures 4A, B). Given the importance of the liver in insulin metabolism, we further analyzed the hepatic lipid profiles of different groups to investigate whether lipid molecules are responsible for the effects of 1,25(OH)_2_D_3_ supplementation. At the 8th weeks, the total content of cardiolipins (CL), phosphatidylethanolamines (PE), and DGs was remarkably higher in the PCOS group than in the CON group. In contrast, the contents of phosphatidylglycerols (PG) and lysophosphatidylserines (LPS) decreased in the PCOS group. Supplementation with 1,25(OH)_2_D in PCOS rats decreased the DG content (Figures 4C, D).

4 The contents of six DGs [DG 32:2 (18:2/14:0), DG 34:3 (18:2/16:1), DG 34:2 (16:0/18:2), DG 36:4 (18:2/18:2), DG 36:3 (18:2/18:1) and DG 38:4 (18:1/20:3)] were markedly increased in the PCOS group compared with the control group. 1,25(OH)_2_D_3_ supplementation significantly decreased the DG content [DG 32:2 (18:2/14:0), DG 32:1 (16:1/16:0), DG 34:3 (18:2/16:1), DG 34:2 (16:0/18:2), DG 34:1 (18:0/18:1), DG 36:4 (18:2/18:2), DG 36:3 (18:2/18:1), DG 36:2 (18:1/18:1), DG 38:6 (18:1/20:5) and DG 38:4 (18:1/20:3)] (Figure 4F).

## 4 Discussion

In this study, we found a significant decrease in vitamin D concentration in letrozole-induced PCOS rats compared to that in control rats. The key findings of the present study were the metabolic improvement of 1,25(OH)_2_D_3_ in PCOS rats and the potential regulatory effects of 1,25(OH)_2_D_3_ on hepatic DG content.

PCOS is a heterogeneous disease accompanied by metabolic abnormalities, such as obesity, insulin resistance, dyslipidemia, and endothelial dysfunction. Improvement of metabolic dysfunction is of paramount importance to reduce the risk of metabolic and cardiovascular disorders in patients with PCOS later in life. Additionally, improvement of metabolic dysfunction is beneficial for reproductive function and pregnancy outcomes (Teede et al., 2010). In the current study, we confirmed that letrozole gavage administration could induce PCOS-like phenotypes in rats, including cystic ovarian morphology with a lack of corpora lutea, disturbance of the estrus cycle, and elevated testosterone levels and LH/FSH ratio. Vitamin D deficiency is reportedly associated with metabolic disturbances in PCOS, and the prevalence of vitamin D deficiency among PCOS women (67%–85%) is much higher than among the healthy population (20%–48%) (Thomson et al., 2012; He et al., 2015). However, whether vitamin D supplementation could exert therapeutic effects on PCOS remains controversial. The discordant results of existing studies may be due to the differences in treatment duration, type, vitamin D dosage, and the characteristics of the study participants (Trummer et al., 2018; Guo et al., 2020). In current work, we also found a significant decrease in vitamin D concentration in letrozole-induced PCOS rats compared to in control rats. These results suggest that vitamin D levels are abnormally changed in PCOS. However, the underlying mechanism requires further investigation.

Consequentially, to further explore the roles of vitamin D in PCOS, active 1,25(OH)_2_D_3_ was injected into PCOS rat models in the present study, which reduced the weight, increased insulin sensitivity, and ameliorated lipid disturbances in PCOS rats. Previous data suggested that approximately 50% of women with PCOS are overweight or obese, and excess weight aggravates metabolic and reproductive disorders in PCOS (Glueck and Goldenberg, 2019), and our results were consistent with the previous observation. It has been reported that, excessive accumulation of adipose tissue can immobilize fat-soluble vitamin D and induce oxidative stress and inflammation in PCOS, which interferes with vitamin D metabolism (Helal et al., 2021; Mancini et al., 2021). In current work, we observed that letrozole-treated rats exhibited a significant increase in body weight and a decrease in serum 25(OH)D concentration; these results are consistent with previous observations (Grzesiak et al., 2021). Moreover, a previous study confirmed amelioration of weight gain after a subcutaneous injection of 1,25(OH)_2_D_3_ (Azhar et al., 2021). Furthermore, the results of randomized, placebo-controlled clinical trials conducted in women with PCOS found that vitamin D supplementation led to metabolic benefits (Foroozanfard et al., 2017; Javed et al., 2019). Javed et al. (2019) Found a modest reduction in homeostatic model assessment-insulin resistance (HOMA-IR) in women with PCOS who were orally administered vitamin D (3200 IU/day) for 3 months. Foroozanfard et al. (2017) Conducted a trial in 90 insulin-resistant patients with PCOS where the participants were allocated to take either 4000 or 1000 IU of vitamin D per day or placebo for 3 months. They observed reduced fasting plasma glucose levels, HOMA-IR, serum triglycerides levels, and total-, LDL-, and total/HDL-cholesterol levels in the high-dose vitamin D group. In PCOS rats, we also observed a decrease in blood glucose levels at 15 min, but a more significant increase in blood glucose levels at 60 min during the OGTT, which indicates altered patterns of glucose metabolism. Furthermore, we found reduced HDL-c levels and elevated LDL-c levels in PCOS rats. These suggest that treatment with 1,25(OH)_2_D_3_ in a letrozole-induced PCOS rat model could prevent weight gain, increase HDL-c levels, improve insulin sensitivity, and decrease postprandial blood glucose levels. Our results are consistent with those of previous studies.

Insulin resistance plays a key role in the onset and progression of PCOS and DG is reportedly associated with insulin-related signaling pathways and is a predictor of insulin resistance (Kumashiro et al., 2011; Finck and Hall, 2015; Jayasinghe et al., 2019). Previous studies on other metabolic diseases, such as obesity and non-alcoholic fatty liver disease, have indicated that the ectopic accumulation of lipids is one of the main causes of insulin resistance in non-adipose tissues. In a letrozole-induced PCOS model, we found that ITT reduced the hypoglycemic effect of insulin, which could be ameliorated by treatment with 1,25(OH)_2_D_3_. Furthermore, the liver is an important target organ of insulin; However, how lipid content is affected in such a model and whether 1,25(OH)_2_D_3_ has regulatory effects remain poorly understood. A previous study conducted using obese rat models showed that insulin resistance is associated with increased DG concentrations (Turinsky et al., 1990). Assessment of liver biopsy samples from obese individuals also revealed that DG content was responsible for the development of insulin resistance (Kumashiro et al., 2011). Multiple studies have suggested that elevated DG levels may impair insulin action in fatty liver disease (Finck and Hall, 2015). In the liver, DGs have been proposed to activate protein kinase Cε (PKCε) (Kumashiro et al., 2011); The activated PKCε phosphorylates the insulin receptor Thr1160 in the kinase activation loop and inhibits insulin receptor kinase activity (Petersen et al., 2016). Furthermore, a subcellular fractionation study revealed that sn-1,2-DG located in the liver plasma membrane is responsible for impeding insulin signaling. Measurement of *in vivo* hepatic insulin signaling indicated that the acute knockdown of hepatic DG acyltransferase-2 increases the accumulation of DG, which in turn activates PKCε and induces insulin resistance in the liver (Lyu et al., 2020). Overall, DG plays a key role in regulating hepatic insulin sensitivity, especially during the pathophysiological processes of obesity, diabetes, and non-alcoholic fatty liver disease. Our data established an *in vivo* letrozole-induced PCOS rodent model with insulin resistance and abnormal liver DG levels. In addition, the current results suggest that 1,25(OH)_2_D_3_ could decrease the level of DG in PCOS and also confirmed the positive correlation between DG and ITT-AUC. In summary, changes in hepatic DG might be associated with the anti-obesity and insulin-sensitizing effects of 1,25(OH)_2_D_3_. Further investigation is required to assess possible underlying mechanisms.

The current study had several limitations. First, the serum 1,25(OH)_2_D levels in rats were not measured. Serum 25(OH)D commonly reflects the vitamin D nutrient status (Bouillon et al., 2008); however, it cannot be used to assess the active vitamin D levels after 1,25(OH)_2_D supplementation. Another limitation was the relatively short study duration. Finally, the correlation between 1,25(OH)_2_D and insulin sensitivity did not prove causality, and the mechanism of DG variation that could affect insulin signaling in PCOS was not investigated in the current study.

In conclusion, vitamin D supplementation could prevent obesity, insulin resistance, and dyslipidemia in a letrozole-induced PCOS rat model. Moreover, this study sheds light on the regulatory effects of 1,25(OH)_2_D_3_ on hepatic DG levels, as indicated by the lipidomic analysis. This phenomenon may explain the protective effects of vitamin D on PCOS. Further studies are required to assess the possible mechanisms underlying the DG-modulating effects of vitamin D. The results of the current study may provide novel evidence for the clinical application of vitamin D as a dietary supplement for long-term management of PCOS.

## Data Availability

The original contributions presented in the study are included in the article/supplementary material, further inquiries can be directed to the corresponding author.
